# Biofunctional Carboxymethyl Chitosan Hydrogel Incorporating Hyaluronic Acid and RGD Peptides for Accelerated Wound Repair

**DOI:** 10.3390/gels11100765

**Published:** 2025-09-23

**Authors:** Shuyue Wang, Qing Yang, Jiren Xu, Youshiqi Zhou, Xiaoqing Tian, Wenhui Wu, Jeevithan Elango, Xiaozhen Diao

**Affiliations:** 1 Department of Marine Pharmacology, College of Food Science and Technology, Shanghai Ocean University, Shanghai 201306, Chinawhwu@shou.edu.cn (W.W.); 2 Shanghai Knowhub Technology Co., Ltd., Pudong New Area, Shanghai 201210, China; 3 Key Laboratory of Oceanic and Polar Fisheries, Ministry of Agriculture and Rural Affairs, East China Sea Fisheries Research Institute, Chinese Academy of Fishery Sciences, Shanghai 200090, China; 4 Marine Biomedical Science and Technology Innovation Platform of Lin-Gang Special Area, Shanghai 201306, China; 5 Putuo Branch of International Combined Research Center for Marine Biological Sciences, Zhoushan 316104, China; 6 Department of Biomaterials Engineering, Faculty of Health Sciences, UCAM—Universidad Católica San Antonio de Murcia, Campus de los Jerónimos 135, 30107 Guadalupe, Murcia, Spain; 7 Center of Molecular Medicine and Diagnostics (COMManD), Department of Biochemistry, Saveetha Institute of Medical and Technical Sciences, Saveetha Dental College and Hospitals, Saveetha University, Chennai 600077, India

**Keywords:** carboxymethyl chitosan, hyaluronic acid, RGD peptide, hydrogel, wound repair

## Abstract

Carboxymethyl chitosan (CMC)-based hydrogels have emerged as promising candidates for wound dressing applications due to their excellent biocompatibility and tunable physicochemical properties. In this study, a novel hydrogel functionalized with hyaluronic acid (HA) and RGD peptides (RGD) was fabricated and evaluated for its structural characteristics and wound-healing potential. Using CMC as the base matrix and EDC/NHS as crosslinking agents, four hydrogel variants were fabricated: CMC gel, CMC-HA gel, CMC-RGD gel, and CMC-HA-RGD gel. The preliminary cell compatibility experiment identified the optimal formulation as 1% CMC, 0.9% HA, and 0.02 mg/mL RGD, crosslinked with 1 vol% EDC and 0.05 wt% NHS. Scanning electron microscopy showed a porous architecture (100–400 μm), conducive to fibroblast viability and proliferation. Zeta potential measurements (|ζ| > 30 mV) indicated colloidal stability of the hydrogel system. Fourier-transform infrared spectroscopy confirmed successful crosslinking and integration of HA and RGD via hydrogen bonding and electrostatic interactions, forming a stable three-dimensional network. Thermogravimetric analysis revealed enhanced thermal stability upon HA/RGD incorporation. CCK-8 assays demonstrated significantly improved cell viability with HA/RGD loading (*p* < 0.05), while Ki-67 immunofluorescence confirmed enhanced fibroblast proliferation, with the CMC-HA-RGD gel showing the most pronounced effect. In vitro scratch assay results demonstrated that the CMC-HA-RGD hydrogel dressing significantly enhanced cellular migration compared to other carboxymethyl chitosan-based hydrogel groups (*p* < 0.05). The observed statistically significant improvement in cell migration rate versus controls underscores the distinctive enhancement of synergistic HA and RGD modification in accelerating cellular migration and facilitating wound repair. Collectively, these findings suggest that the CMC-HA-RGD hydrogel possesses favorable physicochemical and biological properties and holds strong potential as an advanced wound dressing for the treatment of chronic and refractory wounds.

## 1. Introduction

The skin is the largest and most functionally complex organ in the human body [1], performing critical physiological functions including barrier protection, immunity, sensation, and thermoregulation [2]. Failure to achieve timely repair or protracted healing of skin injuries can be life-threatening and severely compromise quality of life [3]. Hydrogels, as an advanced class of soft solid materials, are formed through the physical or chemical crosslinking of natural or synthetic polymers, possessing a unique three-dimensional network structure that endows these materials with excellent physicochemical properties and tunability [4]. Owing to its exceptional hydrophilicity, biocompatibility, and mechanical tunability, the hydrogel has been extensively exploited in diverse biomedical applications including drug delivery, absorbable sutures, and injectable biomaterials—and has demonstrated distinctive and significant clinical potential in accelerating wound repair [5,6]. An ideal wound dressing should not only provide physical protection but also actively orchestrate the four overlapping phases of wound repair-hemostasis, anti-inflammatory/antimicrobial modulation, cellular proliferation, and tissue remodeling [7,8].

Chitosan (CS), the second most abundant natural polysaccharide after cellulose [9], is obtained by the partial deacetylation of chitin and consists of alternating N-acetyl-d-glucosamine and d-glucosamine units linked via β-1,4-glycosidic bonds [10,11]. At physiological pH, the primary amine groups are protonated, conferring a unique cationic character that allows CS to interact electrostatically with negatively charged proteins, nucleic acids, and cell-membrane receptors, thereby exhibiting excellent film-forming, hemostatic, and antimicrobial properties [12]. However, the limited solubility of CS at neutral and alkaline pH restricts its broad application [13]. To overcome this drawback, carboxymethylation has been employed to introduce hydrophilic moieties into the CS backbone, yielding carboxymethyl chitosan (CMCS) [14,15]. CMCS retains the favorable biocompatibility, biodegradability, and amphoteric nature (capable of both positive and negative charges) of its parent chitosan while exhibiting markedly enhanced water solubility and pH stability [2,16]. Crucially, this modification does not introduce additional end-group modifications, allowing CMCS to serve directly as a pristine matrix for constructing skin-repair hydrogels [17]. Compared to carboxymethyl chitin (CMC), CMCS preserves a higher density of free amino groups. This facilitates its efficient enzymatic degradation by lysozyme in vivo, yielding lower-toxicity degradation products [18]. Consequently, CMCS demonstrates superior comprehensive performance in promoting wound hemostasis, anti-inflammatory responses, and subsequent tissue remodeling, establishing it as a scaffold material characterized by enhanced wound healing efficacy for fabricating high-performance wound dressing hydrogels [19,20]. Hang et al. fabricated a macroporous PVA/CMC/PEG composite hydrogel via a synergistic freeze-thaw and phase-separation process, leveraging the excellent biocompatibility of poly (vinyl alcohol) (PVA), the abundant carboxyl and hydroxyl groups of carboxymethyl chitosan (CMC), and the porogenic effect of polyethylene glycol (PEG). To further enhance hemostatic performance, zeolitic imidazolate framework-L (ZIF-L) was in situ synthesized within the hydrogel matrix, yielding the PVA/CMC/PEG@ZIF-L composite hydrogel. A rat liver-injury model confirmed that this composite hydrogel exhibits rapid and effective hemostasis [21].

Hyaluronic acid (HA) is a naturally produced linear high-molecular-weight polymer, classified as a glycosaminoglycan, and constituted by repeating disaccharide units of D-glucuronic acid and N-acetylglucosamine [22]. Owing to its widespread presence across mammalian tissues and its unique structural organization—which includes variable secondary and tertiary configurations—HA exhibits distinctive rheological behavior and a range of biological roles, including the maintenance of tissue homeostasis and regulation of cellular processes [23,24]. As a fundamental component of the extracellular matrix (ECM), HA possesses exceptional hydration and water retention capabilities [25], enabling it to directly recapitulate the physiological microenvironment of the skin and significantly accelerate cell proliferation and migration [26]. Hyaluronic acid (HA) promotes cellular proliferation through CD44 receptor-mediated activation of growth factor signaling pathways (specifically the HB-EGF/EGFR pathway) and key kinase pathways (specifically the MAPK/PI3K pathways) [27,28]. Given the high CD44 expression of the NIH/3T3 cell line and its strong responsiveness to growth factors and serum stimuli, this cell line serves as an ideal in vitro model for validating HA-induced fibroblast proliferation and elucidating the underlying molecular mechanisms [29,30]. Owing to its outstanding biocompatibility, biodegradability, and non-toxicity, HA serves distinct therapeutic and diagnostic roles. Therapeutically, it orchestrates skin and wound repair, drives tissue regeneration, and exerts anti-inflammatory and immunomodulatory effects. Diagnostically, its expression patterns and molecular interactions are exploited for tumor detection and monitoring [31,32]. Existing research has demonstrated that HA promotes the proliferation of NIH-3T3 fibroblasts while inhibiting scar formation and fibrotic progression during the early stages of healing [22,33]. When HA is further engineered into hydrogel form, its three-dimensional interconnected porous network (pore size 50–200 μm). This pore size range is selected to facilitate fibroblast infiltration, proliferation, and collagen deposition, while simultaneously promoting vascular ingrowth and capillary formation—critical processes for successful tissue integration and regeneration [34,35]. Furthermore, the open porous structure supports efficient nutrient diffusion and metabolic exchange, essential for maintaining cellular viability in vitro and in vivo [36,37]. It not only mimics the topological structure of the native ECM but also incorporates RGD peptide binding sites via covalent grafting. Specifically, this functionalization is achieved through a chemical reaction between functional groups on the HA chain (such as carboxyl and hydroxyl groups) and terminal amino groups of the RGD peptide, resulting in stable covalent linkages that anchor the RGD motifs to the hydrogel scaffold. This design provides a more favorable microenvironment for cell adhesion, nutrient diffusion, and subsequent tissue remodeling [32,38,39]. Weiyi et al. constructed a light-responsive supramolecular polysaccharide hydrogel by exploiting host-guest interactions between azobenzene and β-cyclodextrin moieties grafted onto hyaluronic acid chains. Reversible trans-cis isomerization of the azobenzene units under alternating wavelengths allowed dynamic modulation of the network crosslink density: UV irradiation induced hydrogel relaxation and triggered rapid, localized release of epidermal growth factor (EGF) directly within the wound bed. In a murine full-thickness skin-defect model, this spatiotemporally controlled EGF delivery significantly enhanced granulation-tissue formation, elevated growth-factor levels, and accelerated angiogenesis, ultimately restoring the anatomical structure and physiological function of the damaged skin. The study thus introduces an intelligent supramolecular hydrogel platform with considerable potential for advanced wound-healing applications [40].

The RGD (Arg-Gly-Asp) tripeptide is a minimal cell-adhesive motif that specifically recognizes and binds integrin receptors such as αvβ3 and α5β1 on the cell membrane [41,42]. When RGD is incorporated into wound-healing hydrogels, it generates a biomimetic, extracellular-matrix-like interface [32]. This RGD-integrin interaction markedly enhances fibroblast adhesion, spreading, and proliferation, thereby accelerating granulation-tissue formation, while the hydrogel’s three-dimensional porous architecture cooperatively directs ordered cell migration and reduces scar formation, ultimately enabling rapid and high-quality skin regeneration [11,43,44].

This study pioneers the synergistic integration of HA and RGD polypeptides within a CMC hydrogel matrix. By integrating HA and the RGD peptide within a hydrogel matrix, this study develops a three-dimensional, functionalized dressing that overcomes the inherent limitations of conventional wound-care materials. This approach possibly addresses the pivotal challenges of microenvironmental imbalance, impaired cellular migration, and suboptimal tissue regeneration, thereby demonstrating substantial potential to accelerate the wound-healing process.

## 2. Results and Discussion

### 2.1. Fabrication of CMCS Hydrogels

**Figure 1:**
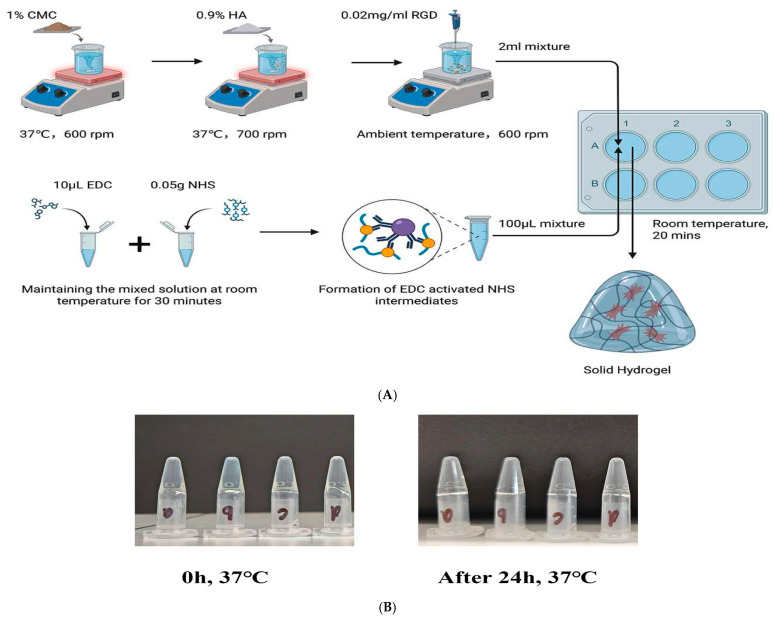
Fabrication process of CMCS hydrogels. The samples were plated in a six-well plate as follows: well A1: CMC hydrogel; well A2: CMC-HA hydrogel; well A3: CMC-RGD hydrogel; well B1: CMC-HA-RGD hydrogel; wells B2 and B3: empty. (**A**) Gelling stability of CMC hydrogel after 24 h at 37 °C (**B**) (a): CMC hydrogel, (b): CMC-HA: CMC hydrogels with 0.9% HA, (c): CMC-RGD hydrogel: CMC hydrogels with 0.02 mg/mL RGD, (d): CMC-HA-RGD: CMC hydrogels with 0.9% HA and 0.02 mg/mL RGD.

As illustrated in Figure 1A, the CMCS-based hydrogel was constructed using the classic EDC/NHS chemical crosslinking method [45]. Specifically, EDC (1-ethyl-3-(3-dimethylaminopropyl) carbodiimide) and NHS (N-hydroxysuccinimide) first synergistically interacted at room temperature to form an activated NHS ester intermediate [46]. This intermediate subsequently underwent an amidation reaction with the carboxyl and amino groups on the CMCS molecular chains, thereby inducing intermolecular crosslinking and ultimately forming a solid hydrogel with a three-dimensional network structure [32]. This process, which requires neither high temperatures nor strong alkaline conditions, proceeds under mild reaction conditions favorable for preserving the bioactivity of HA and RGD. As shown in Figure 1B, after incubating the four sample groups at 37 °C for 24 h, all groups successfully formed stable, non-flowing solid hydrogels, indicating that this formulation (1% CMCS + 0.9% HA + 0.02 mg/mL RGD) possesses excellent gelation capability within the EDC/NHS crosslinking system. Furthermore, no phase separation or collapse was observed, suggesting that the incorporation of HA and RGD did not interfere with the CMCS crosslinking process but potentially enhanced the stability of the gel structure through physical entanglement or hydrogen bonding. These results confirm the feasibility and reproducibility of this method for constructing functional three-dimensional scaffolds.

### 2.2. Scanning Electron Microscopy

**Figure 2:**
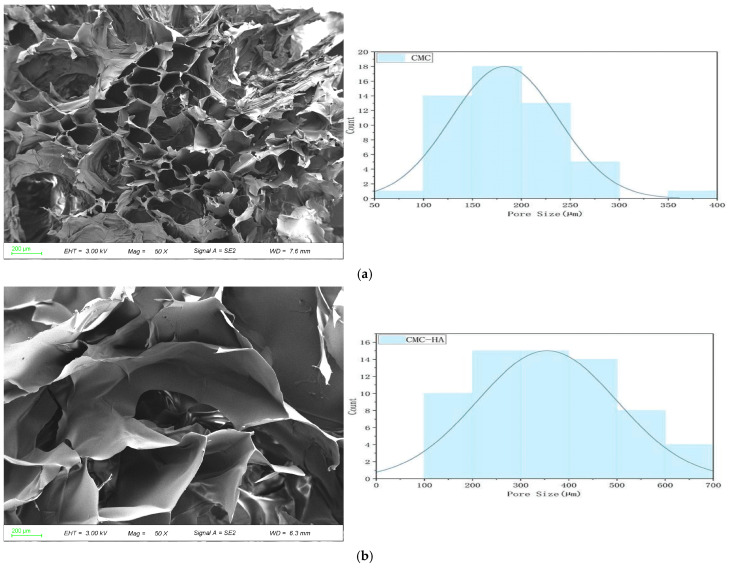

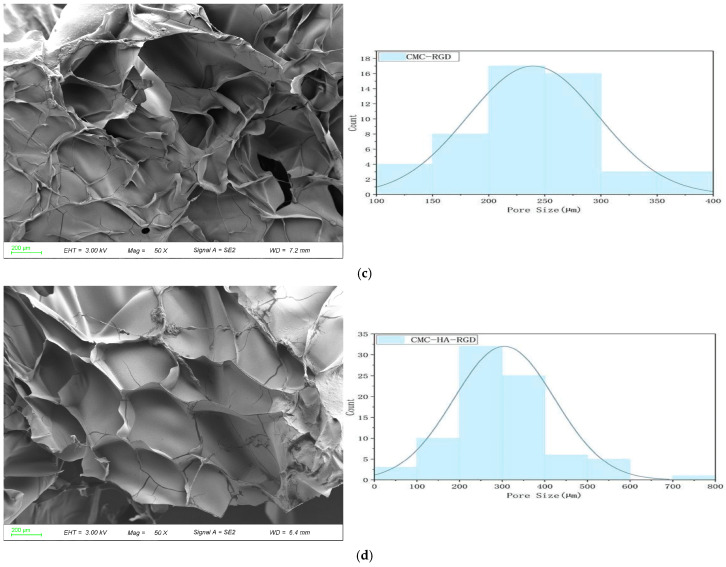
SEM microscopic images of CMCS hydrogels (Freeze-dried). Scale bars 200 µm (Mag = 50×, WD = 6.4 mm). (**a**) CMC hydrogel, (**b**) CMC-HA: CMC hydrogels with 0.9% HA, (**c**) CMC-RGD hydrogel: CMC hydrogels with 0.02 mg/mL RGD, (**d**) CMC-HA-RGD: CMC hydrogels with 0.9% HA and 0.02 mg/mL RGD.

The micro-morphological SEM results (Figure 2) revealed that all four formulations exhibited a typical three-dimensional (3D) porous network structure with normally distributed pore sizes, where the primary peak fell within the range of 100–400 μm. This pore size distribution provided a favorable structural foundation for NIH/3T3 fibroblast migration and proliferation. The pure CMCS hydrogel (a) displayed a pore size peak between 150–250 μm, featuring a uniform and well-interconnected pore structure. This morphology offers homogeneous initial adhesion sites and a localized microenvironment for fibroblasts, facilitating early cell spreading and migration. Upon the incorporation of HA (b), the average pore size significantly increased, and the primary peak shifted upwards to 200–400 μm. These larger pores provide ample volume for the further expansion and fusion of cell clusters within the 3D space. When the RGD peptide was incorporated (c), the primary peak was located at 200–250 μm. The increase in pore size was intermediate between that of CMCS and CMCS-HA, indicating RGD’s moderate regulatory effect on the network structure. Following the synergistic integration of both HA and RGD (d), the pore size distribution exhibited a continuous multiscale characteristic. Micropores (~100 μm) ensured early cell anchoring, medium-sized pores (200–300 μm) supported cell proliferation and extracellular matrix (ECM) deposition, while macropores (>350 μm) accommodated later tissue expansion. Overall, the composite hydrogel exhibited a highly heterogeneous porous architecture with interconnected pores across a broad size range. Such a structure is particularly favorable for fibroblast infiltration and extracellular matrix deposition, thereby providing a supportive microenvironment for wound healing applications.

SEM images of CMC/HA-DA hydrogel prepared by Longlong Cui and other researchers show that the hydrogel had a porous three-dimensional network structure. This structure not only effectively absorbs water and wound exudate but also provides channels for the exchange of oxygen, carbon dioxide, and nutrients, while creating space for cell migration, proliferation, and tissue remodeling [47]. Therefore, the porous three-dimensional structure of hydrogel is the key factor affecting its performance, which significantly affects the cell activity [48,49].

### 2.3. Physicochemical Properties

**Figure 3:**
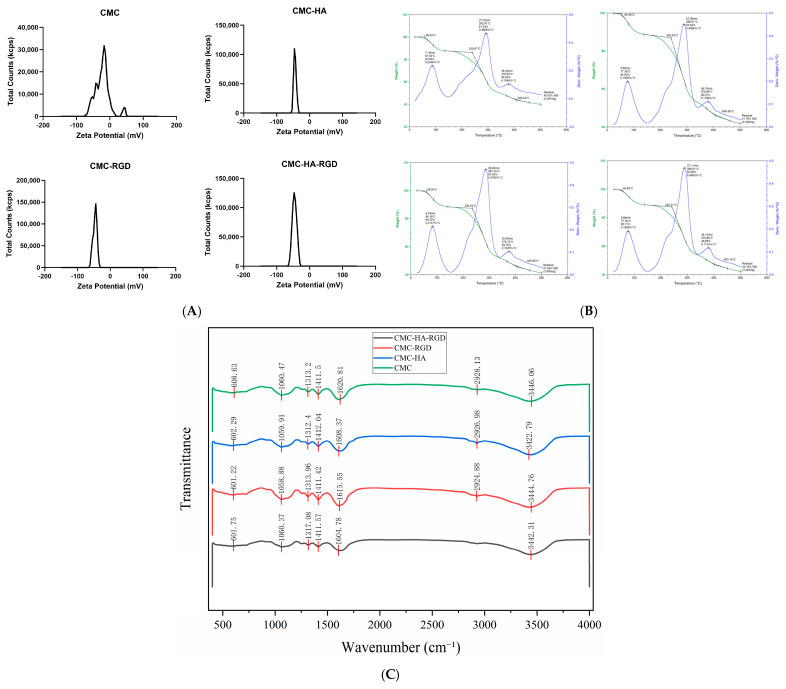
Physicochemical properties of CMCS hydrogels. Zeta potential (pre-crosslinked precursor) (**A**), Thermal degradation profiles (Freeze-dried) (**B**) (thermogravimetric (TG, green solid line) and derivative thermogravimetry (DW, blue solid line)) and FTIR spectra (Freeze-dried) (**C**). CMC hydrogel, CMC-HA: CMC hydrogels with 0.9% HA, CMC-RGD hydrogel: CMC hydrogels with 0.02 mg/mL RGD, CMC-HA-RGD: CMC hydrogels with 0.9% HA and 0.02 mg/mL RGD.

Zeta potential (ζ) is a key indicator for characterizing the stability of colloidal dispersion systems and their surface charge properties. It reflects the charged state at the hydrogel surface or within its internal network structure. Generally, a ζ potential magnitude exceeding 30 mV indicates good stability [50,51,52]. Zeta potential measurements (Figure 3A) revealed that the values ζ of all four formulations exceeded −30 mV, indicating sufficient surface charge density to generate strong electrostatic repulsion, thereby effectively inhibiting particle aggregation and ensuring long-term colloidal stability. The pristine CMC sample exhibited minor secondary peaks, suggesting an inhomogeneous charge distribution, most likely attributable to trace metal ion contaminants or incomplete dissolution of CMC. Upon incorporation of either HA or RGD, these secondary peaks disappeared; both CMC-HA and CMC-RGD displayed single, symmetric, and sharp distributions with unaltered full-width at half-maximum, confirming excellent compatibility among components and the absence of secondary aggregation. The ternary CMC-HA-RGD system retained the same characteristics, further corroborating nanoscale uniformity and high stability. Such robust colloidal behavior enables the formation of a homogeneous drug/growth-factor release interface within wounds, minimizes foreign-body responses caused by particle agglomeration, and provides a reliable physicochemical foundation for subsequent cell infiltration and efficient healing of chronic wounds. Similar to the study by Kanjana et al., ζ potential measurements were employed to assess the stability after three years of storage for tamarind seed kernel xyloglucan hydrogels incorporated with chitosan, prepared at varying ratios. The ζ potential confirmed that the hydrogel with a xyloglucan-chitosan ratio of 1.5:1 exhibited greater stability after storage compared to the 4:1 ratio [53].

Thermogravimetric (TG) curves (Figure 3B) revealed a typical two-stage weight loss behavior for all hydrogels: (1) A minor mass loss of approximately 4–7% between 30–150 °C, attributed to the volatilization of adsorbed and bound water [54,55]; (2) A major degradation stage from 200–450 °C originating from the scission and carbonization of CMC molecular backbones, HA saccharide chains, and RGD peptide chains. All four groups exhibited initial significant degradation temperatures exceeding 200 °C, indicating their potential tolerance for high-temperature sterilization. Specifically, the pure CMC group initiated significant degradation at 239.9 °C with a char residue of 40.03% at 500 °C, confirming the inherent thermal stability of CMC. Upon incorporation of HA alone, the initial degradation temperature increased to 240.53 °C while the char residue at 500 °C rose to 41.76%, suggesting that the hydrogen-bonding network formed between HA and CMC moderately elevated the thermal decomposition threshold. For the RGD-modified group, the initial degradation temperature was 236.03 °C with 41.29% char residue at 500 °C, demonstrating stabilization effects comparable to the HA group. This implies that RGD similarly enhances system stability through electrostatic and hydrogen-bonding interactions. Notably, the CMC-HA-RGD synergistic group displayed further improved performance: an initial degradation temperature of 242.31 °C and char residue of 42.16% at 500 °C, significantly surpassing other groups. This phenomenon is ascribed to the multifaceted physicochemical crosslinking network formed between the polyhydroxy structure of HA, RGD peptide chains, and CMC, which cooperatively enhances thermal decomposition resistance. Consequently, this network provides fundamental support for high-temperature sterilization processes and long-term storage stability.

Yingchun et al. prepared a 37 °C-sensitive hydrogel loaded with the active substance dihydromyricetin (DHM) using poloxamer, chitosan, and hyaluronic acid for hemostasis, and thermogravimetric analysis was also used to determine the thermal stability of the hydrogel. The experimental results showed that DHM first lost approximately 1% of its weight at 130 °C due to the removal of adsorbed/bound water, then started to melt at 252 °C with continuous decomposition, and completely lost weight at 550 °C. In contrast, the initial weight loss temperature of the hydrogel containing DHM increased from 192 °C to 222 °C, indicating that the addition of DHM significantly improved the thermal stability of the hydrogel [56].

Fourier Transform Infrared (FTIR) spectroscopy was employed to analyze the secondary structural evolution of hydrogels pre- and post-formation [4], characterizing the carboxymethyl chitosan (CMC) matrix and its composites incorporating HA, RGD, and the HA-RGD synergistic system (Figure 3C, Table 1). Spectral analysis revealed that all four sample groups retained characteristic CMC skeletal absorption peaks (C-O-C, C-C stretching vibrations) within the 608–1060 cm^−1^ region, confirming structural integrity of the CMC network. Significant band displacements were induced upon incorporation of HA and RGD components: The CMC-HA composite exhibited a redshift of the amide I band from 1620.8 cm^−1^ to 1608.4 cm^−1^, indicative of electrostatic/hydrogen-bonded complex formation between HA carboxylate anions (-COO^−^) and CMC protonated amino groups (-NH_3_^+^). Conversely, the CMC-RGD system demonstrated a slight blueshift to 1615.6 cm^−1^, corroborating hydrogen bonding interactions and potential amidation between RGD carboxyl/peptide functionalities and CMC. The ternary CMC-HA-RGD composite manifested a more pronounced amide I band descent to 1604.8 cm^−1^, evidencing the construction of a highly crosslinked network via hydrogen bonding-electrostatic synergy. Within the aliphatic C-H stretching region (2920–2930 cm^−1^), the characteristic -CH_2_- vibration at 2928 cm^−1^ observed in pristine CMC underwent minor hypsochromic shifts with attenuated intensity in CMC-HA (2926 cm^−1^) and CMC-RGD (2924 cm^−1^), suggestive of restricted molecular chain mobility. Notably, complete spectral suppression of this peak occurred in the CMC-HA-RGD system, attributable to a high-density hydrogen bonding-electrostatic network formed between HA polyhydroxy moieties, RGD guanidinium/carboxyl groups, and CMC hydroxyl/amino functionalities. This network severely constrains aliphatic segmental motion, culminating in the disappearance of C-H stretching vibrations due to hydrogen bond shielding. Collectively, characteristic peak displacements, bandwidth alterations, and the attenuation of the 2920 cm^−1^ peak attest to the formation of a dense, homogeneous hydrogen bond-electrostatic dual-crosslinked network within the CMC-HA-RGD system, establishing the molecular basis for controlled drug/cell factor delivery and chronic wound repair applications.

### 2.4. Cell Proliferation

**Figure 4:**
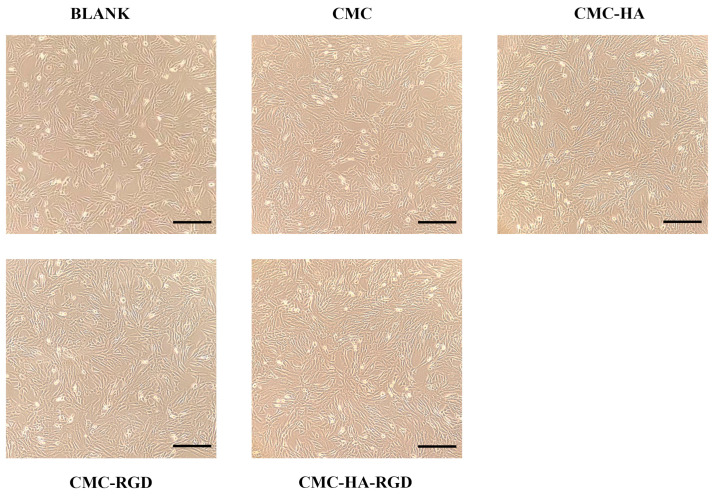
NIH/3T3 cells images of adding CMCS hydrogels (fully cross-linked, hydrated solid hydrogels). Control: only medium and cells, CMC hydrogel, CMC-HA: CMC hydrogels with 0.9% HA, CMC-RGD hydrogel: CMC hydrogels with 0.02 mg/mL RGD, CMC-HA-RGD: CMC hydrogels with 0.9% HA and 0.02 mg/mL RGD. Crosslinker: 1 vol% EDC and 0.05 wt% NHS. Scale bar: 100 μm.

Micro-morphological observations (Figure 4) directly illustrate the adhesion and proliferation of NIH/3T3 fibroblasts on the various hydrogel surfaces. In the control group (a, medium only), cells were sparse and exhibited an elongated spindle shape. On pure CMCS hydrogels (b), cell density increased modestly; the cells remained spindle-shaped with limited spreading, indicating a mild, intrinsic pro-adhesive effect of CMCS. In the CMCS-HA group (c), markedly higher cell numbers were observed, and cells adopted a polygonal morphology, demonstrating that 0.9 wt% HA enhances cell spreading and proliferation by establishing a hydrated, ECM-like microenvironment. CMCS-RGD hydrogels (d) supported greater cell coverage than the control but slightly lower than CMCS-HA; cells appeared markedly flattened, confirming the bioactivity of 0.02 mg/mL RGD peptide in promoting integrin-mediated adhesion. The CMC-HA-RGD combination (e) yielded the highest cell density, with cells reaching near-confluency and displaying a highly extended, polygonal morphology and tight intercellular connections. These results substantiate that the synergistic integration of HA and RGD maximizes cellular adhesion, proliferation, and spreading, thereby providing a robust cytological foundation for subsequent tissue regeneration.

**Figure 5:**
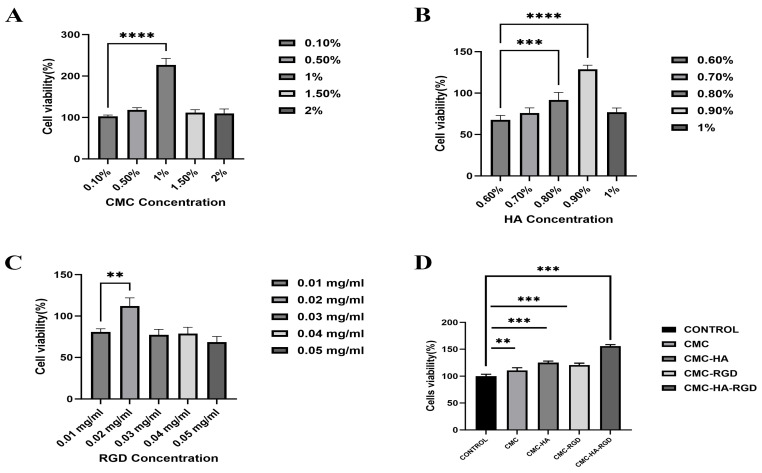
CCK-8 images of CMC concentration selection (**A**) (*p* < 0.001): 0.1, 0.5, 1, 1.5, and 2 wt%; HA concentration selection (**B**) (*p* < 0.001): 0.6, 0.7, 0.8, 0.9, and 1 wt% HA; RGD concentration selection (**C**) (*p* < 0.01): 0.01, 0.02, 0.03, 0.04, and 0.05 mg/mL. CCK-8 images of CMCS hydrogels (fully cross-linked, hydrated solid hydrogels) (**D**) (*p* < 0.01)—Control: only medium and cells, without CMC hydrogel, CMC-HA: CMC hydrogels with 0.9% HA, CMC-RGD hydrogel: CMC hydrogels with 0.02 mg/mL RGD; CMC-HA-RGD: CMC hydrogels with 0.9% HA and 0.02 mg/mL RGD. Crosslinker: 1 vol% EDC and 0.05 wt% NHS. All CCK-8 assays were performed with a sample size of *n* = 3 independent replicates per group. * Denotes a statistical significance between samples: ** (*p* < 0.01); *** (*p* < 0.001); **** (*p* < 0.0001). Statistical test methods: one-way ANOVA and multiple comparison tests. Error bars in all graphs represent the standard deviation (SD).

As shown in Figure 5, CCK-8 assays systematically elucidated the dose-dependent effects of each component on NIH/3T3 fibroblast viability and were consequently used to define the optimal hydrogel formulation. Figure 5A shows that cell viability peaked at a CMCS concentration of 1 wt%; this value was therefore selected to ensure adequate mechanical integrity while minimizing cytotoxicity. Figure 5B demonstrates that 0.9 wt% HA yielded the highest viability, significantly surpassing both lower and higher concentrations, and was thus adopted as the HA loading. Figure 5C indicates that 0.02 mg/mL RGD peptide maximized viability, whereas further increases induced a slight decline; accordingly, 0.02 mg/mL was retained for the final composition.

Hydrogels prepared with these optimized concentrations were subsequently evaluated for cytocompatibility (Figure 5D). Blank controls (culture medium only) were normalized to 100% viability, representing baseline proliferation. Pure CMCS hydrogels exhibited marginally higher viability, suggesting an intrinsic, mild proliferative effect of CMCS. Incorporation of either HA or RGD further elevated viability in CMCS-HA and CMCS-RGD groups, corroborating the capacity of HA to modulate microenvironmental hydration and of RGD to facilitate integrin-mediated adhesion. The CMC-HA-RGD group achieved the highest viability, significantly exceeding all other formulations (*p* < 0.001), demonstrating a synergistic enhancement that collectively provides a robust cellular foundation for efficient tissue regeneration.

Ebhodaghe et al., in their systematic review, pointed out that carboxymethyl chitosan (CMC) hydrogels can serve as three-dimensional scaffolds for fibroblast adhesion and proliferation, and significantly enhance the survival rate and collagen secretion level of NIH/3T3 and mouse primary fibroblasts in various skin trauma models [57]. Kanikireddy et al. demonstrated that the composite hydrogel with CMC as the matrix, when co-cultured with human skin fibroblasts in vitro for 7 days, led to a 2.8-fold increase in cell density and a significant rise in the Ki-67 positive rate, confirming its good cytocompatibility and proliferation-promoting effect [58]. Zhao et al. reported that when CMC/poloxamer thermosensitive hydrogel was applied to a mouse full-thickness skin defect model, histological results showed that the hydrogel induced a large number of fibroblasts to infiltrate on day 14, accompanied by the ordered deposition of collagen fibers, suggesting that the CMC network provides a favorable microenvironment for fibroblast migration, proliferation and ECM remodeling [56,59,60].

### 2.5. Fluorescence Staining

**Figure 6:**
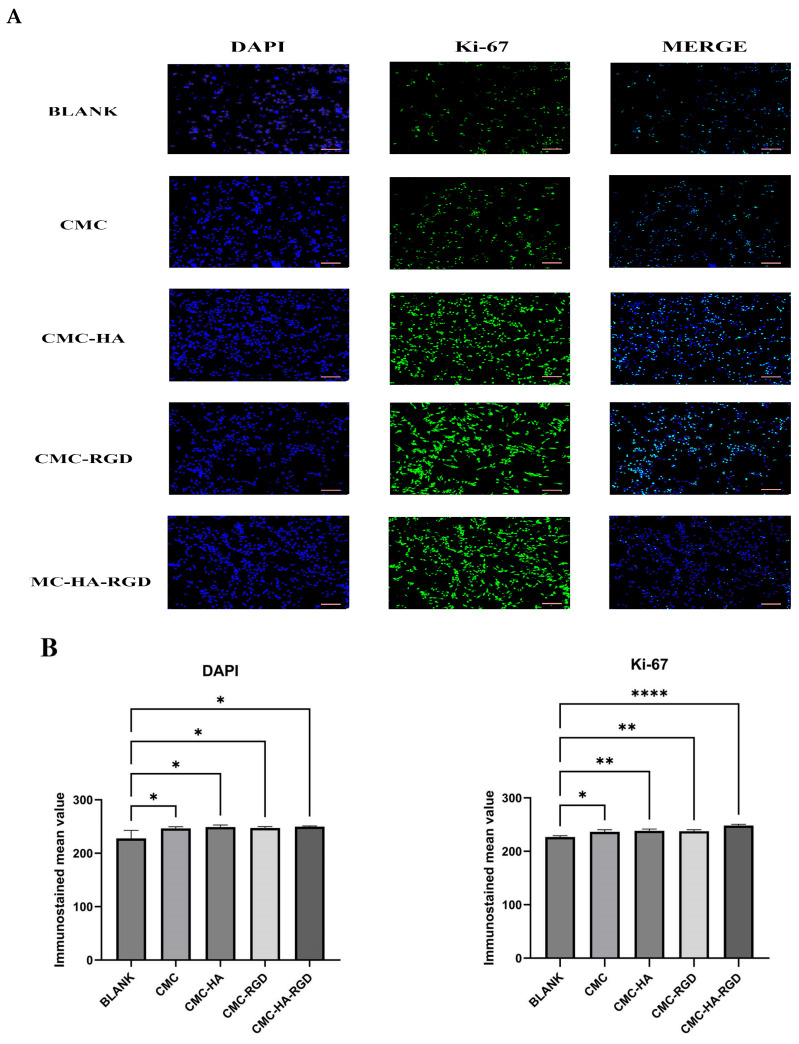
Immunofluorescence micrographs of Ki-67 in NIH/3T3 fibroblasts cultured on CMCS hydrogels (fully cross-linked, hydrated solid hydrogels) (**A**) (*p* < 0.05). Immunofluorescence mean value of NIH/3T3 fibroblasts cultured on CMCS hydrogels (**B**) (*p* < 0.05). Blank: only medium and cells, CMC hydrogel, CMC-HA: CMC hydrogels with 0.9% HA, CMC-RGD hydrogel: CMC hydrogels with 0.02 mg/mL RGD, CMC-HA-RGD: CMC hydrogels with 0.9% HA and 0.02 mg/mL RGD. All statistical analyses were performed using GraphPad Prism software. Comparisons among groups were carried out using one-way analysis of variance (ANOVA) followed by post hoc multiple comparison tests. Data are presented as mean ± standard deviation (SD). Each experimental condition was tested in at least three independent replicates (*n* = 3). Error bars in all graphs represent the standard deviation (SD). Statistical significance was indicated as follows: * (*p* < 0.05); ** (*p* < 0.01); **** (*p* < 0.0001). Scale bar: 100 μm.

Immunofluorescence results (Figure 6A) showed that in Ki-67 immunofluorescence staining, green fluorescence represented the expression of Ki-67 protein (positive signal), while the blue DAPI-stained cell nuclei, and the Merge image displays their co-localization. The blank control group (BLANK) exhibited sparsely distributed round cells with weak Ki-67 signal intensity, while the CMC-only group showed limited cell spreading and sporadic Ki-67-positive signal. Notably, hydrogels modified with either HA or RGD (CMC-HA or CMC-RGD groups) induced increased cell density accompanied by enhanced Ki-67 fluorescence intensity. Among these, the ternary CMC-HA-RGD composite displayed the strongest Ki-67-positive signal, suggesting that this hydrogel formulation is more conducive to cell proliferation. Quantitative analysis of the bar graph (Figure 6B) revealed that the Ki-67 fluorescence intensity in the CMC-HA-RGD group was significantly higher than in all other groups (*p* < 0.001). DAPI fluorescence intensity showed a significant difference among groups (*p* < 0.05), further confirming that the CMC-HA-RGD hydrogel significantly promoted cell proliferation. These differential effects emerged within 24 h, indicating that synergistic HA/RGD interactions rapidly activate the cell cycle, promoting the proliferation and spreading of NIH/3T3 fibroblasts [31,56].

### 2.6. Cell Migration

**Figure 7:**
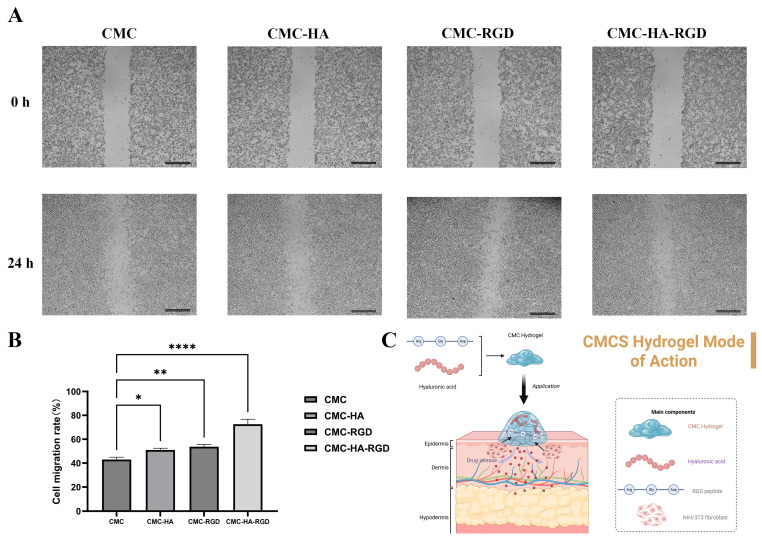
Scratch assay micrographs of NIH/3T3 fibroblasts cultured on CMCS hydrogels (fully cross-linked, hydrated solid hydrogels) (**A**) (*p* < 0.05). Cell migration rate of NIH/3T3 fibroblasts cultured on CMCS hydrogels (**B**). CMCS hydrogels mode of action (**C**). CMC hydrogel, CMC-HA: CMC hydrogels with 0.9% HA, CMC-RGD hydrogel: CMC hydrogels with 0.02 mg/mL RGD, CMC-HA-RGD: CMC hydrogels with 0.9% HA and 0.02 mg/mL RGD. All statistical analyses were performed using GraphPad Prism software. Comparisons among groups were carried out using one-way analysis of variance (ANOVA) followed by post hoc multiple comparison tests. Data are presented as mean ± standard deviation (SD). Each experimental condition was tested in at least three independent replicates (*n* = 3). Error bars in all graphs represent the standard deviation (SD). Statistical significance was indicated as follows: * (*p* < 0.05); ** (*p* < 0.01); **** (*p* < 0.0001). Scale bar: 100 μm.

As illustrated in Figure 7A, the initial scratch widths (0 h) were comparable across all four hydrogel groups. Within 24 h, the wound closure followed the order: CMC-HA-RGD > CMC-RGD ≈ CMC-HA > CMC. Quantitative analysis in Figure 7B revealed that compared to the CMC group, both CMC-HA and CMC-RGD groups exhibited statistically significant enhancements in cellular migration rates (*p* < 0.05), indicating that HA or RGD modification promotes cell migration. Notably, the CMC-HA-RGD group demonstrated significantly higher migration rates than all other groups (*p* < 0.001), suggesting a synergistic enhancement through dual modification with HA and RGD. Given that cell migration is pivotal in wound repair, these findings suggest that these hydrogels—particularly CMC-HA-RGD—may contribute positively to wound repair by accelerating cell migration. Figure 7C illustrates the mode of action of CMC-based hydrogels in facilitating skin wound healing. The hydrogel, composed of carboxymethyl chitosan (CMC), hyaluronic acid (HA), and RGD peptide, is applied to the wound site. Upon application, the hydrogel system either releases bioactive components or maintains a moist, biocompatible microenvironment. Hyaluronic acid contributes as a supportive matrix, while the RGD peptide via its Arg-Gly-Asp sequence mediates specific adhesion and migration of cells like NIH/3T3 fibroblasts. Simultaneously, the CMC hydrogel network acts as a scaffold to retain moisture and sustain the availability of these bioactive factors. Together, these components synergistically created an optimal microenvironment that promoted fibroblast migration into the wound area, thus highlighting the hydrogel’s efficacy as a wound dressing. Martorana et al. developed an injectable hydrogel system using two hyaluronic acid derivatives. Their scratch assay revealed that at 24 h, the wound closure rate was less than 20% in untreated controls, whereas experimental groups containing either F0.30 or AgF0.30 materials achieved 35–49%. By 48 h, near-complete wound closure was observed in treatment groups, while controls remained below 55% closure. These results confirm that the hydrogel system significantly promoted keratinocyte migration and proliferation (*p* < 0.05), thereby providing compelling evidence for accelerated wound repair [61]. While this study successfully demonstrated the hydrogel’s excellent biocompatibility and its ability to accelerate wound repair, a limitation is the absence of a detailed rheological characterization. Future studies should therefore include a comprehensive analysis of the material’s viscoelastic properties to fully understand the relationship between its mechanical environment and its biofunctional performance.

Based on the in vitro experimental results of the four groups of samples and combined with existing studies, it can be confirmed that CMCS hydrogels can serve as three-dimensional scaffolds for fibroblast adhesion and proliferation to improve cell survival rate, and exhibit good cytocompatibility. Therefore, the fabricated hydrogels had an in vitro biological basis for being used as wound repair dressings.

## 3. Conclusions

This study successfully fabricated carboxymethyl chitosan (CMC)-based hydrogels, including binary composites with HA and RGD peptides (CMC-HA, CMC-RGD), and a ternary composite incorporating both HA and RGD (CMC-HA-RGD). Physicochemical characterization demonstrated that these hydrogels, particularly the CMC-based variants, possess excellent thermal stability (degradation temperature > 200 °C), a homogeneous porous structure (pore size 100–400 μm), and favorable long-term colloidal stability (|ζ| > 30 mV). In vitro cell experiments confirmed the non-cytotoxicity of the hydrogels towards NIH/3T3 fibroblasts at the optimal formulation (1 wt% CMC, 0.9 wt% HA, 0.02 mg/mL RGD). Furthermore, the hydrogels significantly enhanced cellular behavior: increased cell spreading area and elevated Ki-67 positivity were observed within 24 h, indicating effective cell cycle activation. The scratch assay confirmed that the composite hydrogels exhibited a more significant promotive effect on cell migration compared with the pure CMC hydrogel. Notably, the HA- and RGD-co-modified hydrogel demonstrated superior wound repair potential, achieving a 72.5% cell migration rate at 24 h—significantly higher than other groups (*p* < 0.001)—thus exhibiting optimal therapeutic efficacy for wound repair. Based on these outstanding physicochemical properties and bioactivities, the developed CMC-HA-RGD ternary composite hydrogel demonstrates considerable potential as an intelligent dressing for chronic refractory wounds, exhibiting promising clinical translation prospects.

## 4. Materials and Methods

### 4.1. Optimization of CMCS Hydrogels

To optimize raw materials for hydrogel fabrication, formulations incorporating varying concentrations of carboxymethyl chitosan (CMC; Sigma-Aldrich, Shanghai, China; CAS: 83512-85-0; carboxymethylation ≥ 80%, MW: 543.5 g/mol, viscosity: 200–300 cps), hyaluronic acid (HA, medium-high molecular-weight grade, with a molecular-weight of 1.0–1.3 MDa, Macklin, Shanghai, China; CAS: 9004-61-9; purity ≥ 97%), and RGD peptide (Sigma-Aldrich, Shanghai, China) were prepared. Concentration gradients were established as follows: CMC at 0.1, 0.5, 1, 1.5, and 2 wt%; HA at 0.6, 0.7, 0.8, 0.9, and 1 wt%; RGD at 0.01, 0.02, 0.03, 0.04, and 0.05 mg/mL. Mouse embryonic fibroblast NIH/3T3 cells were cultured as per the standard instructions as described in vitro experiment. The cells with a density of 10^4^ were seeded in 96-well plate (96-well culture plate, Labselect, Beijing, China) and cultured with CMC, CMC-HA, CMC-RGD and CMC-HA-RGD for 1 day. Then, the cell viability was quantified using the CCK-8 (Cell Counting kit-8, Lot No. M4839, AbMole, Houston, TX, USA) method based on the manufacturer’s instructions.

### 4.2. Fabrication Method of CMCS Hydrogels

Based on optimal (highest viability) cell viability as shown in Figure 5, the formulation containing 1 wt% CMC, 0.9 wt% HA, and 0.02 mg/mL RGD was selected for hydrogel synthesis. Briefly, 0.1 g CMC (1 wt%) was dissolved in 10 mL distilled water under continuous magnetic stirring at 600 rpm and 37 °C. The solution was subsequently crosslinked by adding a pre-activated mixture of EDC (0.05 g) and NHS (10 μL), which had been pre-mixed at 25 °C for 30 min. The reaction was carried out for 20 min to form the unmodified hydrogel, designated as CMC-Control (a) [62,63]. Functionalized hydrogels were subsequently prepared by blending the base hydrogel with (b) 0.9 wt% HA (CMC-HA gel), (c) 0.02 mg/mL RGD (CMC-RGD gel), and (d) both 0.9 wt% HA and 0.02 mg/mL RGD (CMC-HA-RGD gel) (Figure 1). Following crosslinking, the hydrogels were subjected to multiple washes with phosphate-buffered saline (PBS) and distilled water to remove non-crosslinked and subsequent by-products, including the water-soluble urea derivative of EDC and hydrolyzed NHS esters. The crosslinked hydrogels were freeze-dried for characterization experiments and hydrated solid hydrogels were used for cell culture experiments.

### 4.3. Morphological Analysis of CMCS Hydrogels

The microstructure of freeze-dried samples was examined using a ZEISS Gemini SEM 300 ultra-high-resolution field emission scanning electron microscope (Carl ZEISS Ltd., Oberkochen, Germany). Following freeze-drying, the samples were mounted on conductive adhesive tape and sputter-coated with a 10-nm gold layer using a Quorum SC7620 coater (Quorum Technologies, Lewes, UK) at 10 mA for 45 s. Surface morphology was observed using the same ZEISS Gemini SEM 300 microscope at an 3.0 kV accelerating voltage, 6.3 mm working distance and a magnification of 50×, with a scale bar of 200 μm.

### 4.4. Zeta Potential Measurement of CMCS Hydrogels

Zeta potential was determined using a laser diffraction particle size analyzer (Malvern Zetasizer Pro, Malvern, UK). Prior to measurement, the instrument was preheated for 15 min. All samples (pre-crosslinked liquid precursor) were diluted prior to zeta potential measurement. The stock solutions were diluted 10-fold using ultrapure water that had been filtered through a 0.22 µm membrane. The dedicated zeta potential cuvette was rinsed three times with distilled water and then three times with the sample. The sample was subsequently injected into the cuvette up to the standard calibration mark (ensuring equal liquid levels on both sides), and the cuvette’s filling ports were sealed. The cuvette was placed in the instrument’s sample compartment, and measurements were conducted via the accompanying software. Zeta potential was analyzed at 25 °C in ultrapure water, using the “Carboxymethyl Chitosan” material model and an equilibration time of 120 s.

### 4.5. Thermogravimetric Analysis (TGA) of CMCS Hydrogels

The thermal stability of the CMCS hydrogel was evaluated by simultaneous thermal analysis (STA) using a NETZSCH TG 209 F1 Libra instrument (Selb, Germany). Approximately 5 mg of freeze-dried sample was evenly distributed in an alumina crucible and an empty alumina pan was used as the reference. Measurements were performed under a continuous nitrogen flow (≥99.9%) at a heating rate of 10 °C min^−1^ from 25 °C to 500 °C, during which the thermogravimetric (TG) curve was recorded.

### 4.6. Fourier-Transform Infrared Spectroscopy (FTIR) of CMCS Hydrogels

Fourier transform infrared (FTIR) spectroscopy was performed using a Spotlight 400 spectrometer (L1050050, PerkinElmer Inc., Hopkinton, MA, USA). Spectra were acquired in attenuated total reflection (ATR) mode with approximately 1–2 mg of sample (freeze-dried sample) evenly distributed on the diamond crystal. Data collection ranged from 400 to 4000 cm^−1^ at a resolution of 4 cm^−1^, co-adding 32 scans per spectrum. An air background measurement was recorded prior to sample analysis. Environmental H_2_O and CO_2_ interference were automatically compensated during measurement. All spectra underwent baseline correction and peak deconvolution using PeakFit v4.0 software and OMNIC Specta (SeaSolve software Inc., Framingham, MA, USA).

### 4.7. In Vitro Experiment

Mouse embryonic fibroblast NIH/3T3 cells were provided by Tongji University Cell Bank, Shanghai, China and cultured as per the standard instructions. The cells were cultured in High-glucose DMEM medium (Shanghai Qida Biotechnology Co., Ltd., China) supplemented with 10% (*v*/*v*) fetal bovine serum (FBS) and 1% (*v*/*v*) penicillin-streptomycin at 37 °C under 5% CO_2_ in a humidified incubator (BB 150, Thermo Fisher Scientific, Waltham, MA, USA), with medium changed every 48 h until confluence. Upon reaching 80–90% confluency, cells were passaged using 0.25% trypsin-EDTA (Lanjieko Technology Co., Ltd., Hefei, China), and passages 3–8 were used for subsequent experiments.

### 4.8. Hydrogel Sterilization for Cell Culture

Hydrogel precursor solutions containing varying concentrations of Carboxymethyl chitosan (CMC; 0.1%, 0.5%, 1%, 1.5%, and 2 wt%), hyaluronic acid (HA; 0.6%, 0.7%, 0.8%, 0.9%, and 1 wt%), and RGD peptide (0.01, 0.02, 0.03, 0.04, and 0.05 mg/mL) were prepared and dispensed into 96-well plates (100 μL/well). Four hydrogel (hydrated solid hydrogels) formulations were established: CMCS, CMCS-HA, CMCS-RGD, and CMCS-HA-RGD. Each well received 10 μL of EDC/NHS crosslinking solution, followed by gelation at room temperature. Hydrogels were sterilized under UV light for 1 h using the UV-C germicidal lamp (wavelength 254 nm) integrated within the biosafety cabinet. Following UV treatment, the hydrogels were rinsed three times with phosphate-buffered saline (PBS) (Servicebio Co., Ltd., Wuhan, China) and subsequently used for cell culture experiments.

### 4.9. Proliferation

To assess the proliferative effects of CMCS-based hydrogels (hydrated solid hydrogels, CMC hydrogel, CMC-HA: CMC hydrogels with 0.9% HA, CMC-RGD hydrogel: CMC hydrogels with 0.02 mg/mL RGD, CMC-HA-RGD: CMC hydrogels with 0.9% HA and 0.02 mg/mL RGD. Crosslinker: 1 vol% EDC and 0.05 wt% NHS), NIH/3T3 fibroblasts were cultured and evaluated using the Cell Counting Kit-8 (CCK-8; Lot No. M4839, AbMole, Houston, TX, USA), following the manufacturer’s protocol. The precursor solutions for the four sample groups were first prepared in 5 mL centrifuge tubes. After adding the crosslinking agent, the mixtures were homogenized by pipetting. Subsequently, 100 μL of each solution was quickly transferred into individual wells of a 96-well plate and allowed to stand for 30 min to form solid hydrogels. The hydrogels were then sterilized under UV light for 1 h in a clean bench. Following sterilization, the hydrogels were washed three times with PBS buffer. Finally, cells were seeded onto the hydrogels. NIH/3T3 cells were seeded onto the hydrogels at a density of 1 × 10^4^ cells/well in 100 μL of complete culture medium (High-glucose DMEM medium supplemented with 10% (*v*/*v*) fetal bovine serum (FBS) and 1% (*v*/*v*) penicillin-streptomycin). Control wells contained cells and medium without hydrogel. All conditions were tested in triplicate. Plates were incubated at 37 °C in a humidified atmosphere with 5% CO_2_ for 24 h. After incubation, the medium was aspirated, and the cells were gently washed three times with PBS. Subsequently, 110 μL of serum-free medium containing 10% CCK-8 reagent was added to each well. Plates were incubated in the dark for 2.5 h, and absorbance was measured at 450 nm using a microplate reader (BioTek, Winooski, VT, USA). Relative cell viability was calculated by normalizing absorbance values to blank wells (cells without hydrogel), which were defined as 100% viability.

### 4.10. Ki-67 Immunocytochemistry

Cell proliferation was further assessed by quantifying Ki-67 immunofluorescence intensity in NIH/3T3 fibroblasts. Cells were seeded at a density of 2 × 10^5^ cells per well in 6-well plates and cultured in complete medium for 24 h as described earlier. Subsequently, CMCS hydrogel was added to each well and incubated for an additional 24 h. After treatment, the culture medium was aspirated, and cells were gently rinsed once with phosphate-buffered saline (PBS). Fixation was performed using 1 mL of 4% paraformaldehyde per well for 10–15 min at room temperature. Fixed cells were washed three times with PBS (3–5 min per wash) to remove residual fixative.

Following three PBS washes, nonspecific binding was blocked using immunostaining blocking buffer for 20 min at room temperature. Cells were then incubated overnight at 4 °C with Ki-67 monoclonal mouse antibody (diluted 1:200). After additional PBS washes, samples were incubated for 1 h in the dark with Alexa Fluor 488-conjugated goat anti-mouse IgG (diluted 1:400). Nuclei were counterstained with DAPI (2 μg/mL) for 5 min in the dark. After final washes, coverslips were mounted with anti-fade medium and visualized under a fluorescence microscope. Ki-67 expression was detected as green fluorescence, and nuclei were identified in blue.

### 4.11. Scratch Assay

Three equidistant parallel lines were drawn on the back of 6-well plates as positioning markers. Cells from T25 flasks (Lanjieko Technology Co., Ltd., Hefei, China) were trypsinized, counted, and seeded into the plates at a density of 2 × 10^5^ cells/well. After incubation at 37 °C under 5% CO_2_ until reaching 80–90% confluency, wounds were created in the monolayers on the following day using a sterile 200 μL pipette tip perpendicular to the pre-drawn lines. The wells were gently washed 2–3 times with PBS to remove detached cells, replaced with serum-free medium, and immediately imaged under an inverted microscope to record initial wound areas (0 h). The cells were then treated with CMC, CMC-HA, CMC-RGD, or CMC-HA-RGD hydrogels (hydrated solid hydrogels), followed by incubation for 24 h at 37 °C with 5% CO_2_.

Wound Closure (%) = [(A_0_ − A_t_)/A_0_] × 100%

where A_0_ is the initial wound area (at 0 h), A_t_ is the wound area at the measured time point.

Post-incubation wound repair was documented by microscopy. Residual wound areas were quantified using ImageJ software to assess the impact of materials on cell migration capacity.

### 4.12. Statistical Analysis

All experiments were performed in triplicate, yielding consistent results. Data are presented as mean ± standard deviation (SD) unless otherwise specified. Statistical significance was determined by one-way ANOVA with *p* < 0.05 considered significant, using GraphPad Prism software (v.10.1.2, GraphPad Inc., La Jolla, CA, USA).

## Figures and Tables

**Table 1:** Major peaks observed in FTIR spectra of CMCS hydrogels.

CMC	CMC-HA	CMC-RGD	CMC-HA-RGD	Functional Group
608.63	602.29	601.22	601.75	C-O-C stretching
1060.47	1059.91	1058.88	1060.37	C-C skeletal vibrations
1313.20	1312.40	1313.96	1317.08	C-N stretching/-OH bending
1411.50	1412.04	1411.42	1411.57	-COO^−^ symmetric stretching
1620.81	1608.37	1615.55	1604.78	Amide I band (mainly C=O stretching)
2928.13	2926.98	2924.88		-CH_2_- stretching
3446.06	3422.79	3444.76	3442.31	-OH stretching

FTIR Major peaks (Wavenumbers).

## Data Availability

The raw data supporting the conclusions of this article will be made available by the corresponding authors on request.

## References

[1] Pereira R.F., Bártolo P.J. (2016). Traditional Therapies for Skin Wound Healing. Adv. Wound Care.

[2] Mo Y.H., Wang H., Jin S.H., Peng K.L., Yang Z.M., Li P.W., Chen Y. (2022). Preparation and properties of a fast curing carboxymethyl chitosan hydrogel for skin care. Polym. Test..

[3] Wei Q., Wang Y., Wang H., Qiao L., Jiang Y., Ma G., Zhang W., Hu Z. (2022). Photo-induced adhesive carboxymethyl chitosan-based hydrogels with antibacterial and antioxidant properties for accelerating wound healing. Carbohydr. Polym..

[4] Jeevithan L., Shuyue W., Thomas S., de Val J.E.M.S., Wu W., Elango J. (2025). Stem cell-mediated bone regeneration of marine-derived fibrinolytic compound (FGFC-1) loaded carboxymethyl chitosan hydrogels. Biomed. Pharmacother..

[5] Rajabi M., McConnell M., Cabral J., Ali M.A. (2021). Chitosan hydrogels in 3D printing for biomedical applications. Carbohydr. Polym..

[6] Kim S., Cui Z.-K., Fan J., Fartash A., Aghaloo T.L., Lee M. (2016). Photocrosslinkable chitosan hydrogels functionalized with the RGD peptide and phosphoserine to enhance osteogenesis. J. Mater. Chem. B.

[7] Liang Y., Li Z., Huang Y., Yu R., Guo B. (2021). Dual-Dynamic-Bond Cross-Linked Antibacterial Adhesive Hydrogel Sealants with On-Demand Removability for Post-Wound-Closure and Infected Wound Healing. ACS Nano.

[8] Liu H., Wang C., Li C., Qin Y., Wang Z., Yang F., Li Z., Wang J. (2018). A functional chitosan-based hydrogel as a wound dressing and drug delivery system in the treatment of wound healing. RSC Adv..

[9] Guo Y., Qiao D., Zhao S., Liu P., Xie F., Zhang B. (2024). Biofunctional chitosan-biopolymer composites for biomedical applications. Mater. Sci. Eng. R Rep..

[10] Kim Y., Zharkinbekov Z., Raziyeva K., Tabyldiyeva L., Berikova K., Zhumagul D., Temirkhanova K., Saparov A. (2023). Chitosan-Based Biomaterials for Tissue Regeneration. Pharmaceutics.

[11] Tsai W.-B., Chen Y.-R., Liu H.-L., Lai J.-Y. (2011). Fabrication of UV-crosslinked chitosan scaffolds with conjugation of RGD peptides for bone tissue engineering. Carbohydr. Polym..

[12] Xia G., Lang X., Kong M., Cheng X., Liu Y., Feng C., Chen X. (2016). Surface fluid-swellable chitosan fiber as the wound dressing material. Carbohydr. Polym..

[13] Rani K., Malik A.K., Setia A., Randhave N.V., Verma N., Kumar V., Vaishali, Deshmukh K., Muthu M.S. (2025). Chitosan and its derivatives as nanotheranostics in multiple diseases management: A clinical perspective. Carbohydr. Polym..

[14] Dragostin O.-M., Tatia R., Samal S.K., Oancea A., Zamfir A.S., Dragostin I., Lisă E.-L., Apetrei C., Zamfir C.L. (2020). Designing of Chitosan Derivatives Nanoparticles with Antiangiogenic Effect for Cancer Therapy. Nanomaterials.

[15] Sahariah P., Másson M. (2017). Antimicrobial Chitosan and Chitosan Derivatives: A Review of the Structure-Activity Relationship. Biomacromolecules.

[16] Fonseca-Santos B., Chorilli M. (2017). An overview of carboxymethyl derivatives of chitosan: Their use as biomaterials and drug delivery systems. Mater. Sci. Eng. C.

[17] Shariatinia Z. (2018). Carboxymethyl chitosan: Properties and biomedical applications. Int. J. Biol. Macromol..

[18] Lei M., Huang W., Jin Z., Sun J., Zhang M., Zhao S. (2022). Effect of molecular structure and ionization state on aggregation of carboxymethyl chitosan: A molecular dynamics study. Carbohydr. Polym..

[19] Fan L., Yi J., Tong J., Zhou X., Ge H., Zou S., Wen H., Nie M. (2016). Preparation and characterization of oxidized konjac glucomannan/carboxymethyl chitosan/graphene oxide hydrogel. Int. J. Biol. Macromol..

[20] Kłosiński K.K., Wach R.A., Girek-Bąk M.K., Rokita B., Kołat D., Kałuzińska-Kołat Ż., Kłosińska B., Duda Ł., Pasieka Z.W. (2022). Biocompatibility and Mechanical Properties of Carboxymethyl Chitosan Hydrogels. Polymers.

[21] Yang H., Lan X., Xiong Y. (2022). In Situ Growth of Zeolitic Imidazolate Framework-L in Macroporous PVA/CMC/PEG Composite Hydrogels with Synergistic Antibacterial and Rapid Hemostatic Functions for Wound Dressing. Gels.

[22] Isık S., Taşkapılıoğlu M.Ö., Atalay F.O., Dogan S. (2015). Effects of cross-linked high-molecular-weight hyaluronic acid on epidural fibrosis: Experimental study. J. Neurosurg. Spine.

[23] Wu Y., Zhao S., Wang J., Chen Y., Li H., Li J.-P., Kan Y., Zhang T. (2024). Methods for determining the structure and physicochemical properties of hyaluronic acid and its derivatives: A review. Int. J. Biol. Macromol..

[24] Sionkowska A., Gadomska M., Musiał K., Piątek J. (2020). Hyaluronic Acid as a Component of Natural Polymer Blends for Biomedical Applications: A Review. Molecules.

[25] Gao G., Kim B.S., Jang J., Cho D.-W. (2019). Recent Strategies in Extrusion-Based Three-Dimensional Cell Printing toward Organ Biofabrication. ACS Biomater. Sci. Eng..

[26] Domingues R.M.A., Silva M., Gershovich P., Betta S., Babo P., Caridade S.G., Mano J.F., Motta A., Reis R.L., Gomes M.E. (2015). Development of Injectable Hyaluronic Acid/Cellulose Nanocrystals Bionanocomposite Hydrogels for Tissue Engineering Applications. Bioconjug. Chem..

[27] Bhattacharya D.S., Svechkarev D., Souchek J.J., Hill T.K., Taylor M.A., Natarajan A., Mohs A.M. (2017). Impact of structurally modifying hyaluronic acid on CD44 interaction. J. Mater. Chem. B.

[28] Amorim S., Pashkuleva I., Reis C.A., Reis R.L., Pires R.A. (2020). Tunable layer-by-layer films containing hyaluronic acid and their interactions with CD44. J. Mater. Chem. B.

[29] Park H.K., Lee S.J., Oh J.S., Lee S.G., Jeong Y.I., Lee H.C. (2015). Smart Nanoparticles Based on Hyaluronic Acid for Redox-Responsive and CD44 Receptor-Mediated Targeting of Tumor. Nanoscale Res. Lett..

[30] Wang L., Zhang H., Qin A., Jin Q., Tang B.Z., Ji J. (2016). Theranostic hyaluronic acid prodrug micelles with aggregation-induced emission characteristics for targeted drug delivery. Sci. China Chem..

[31] Bukhari S.N.A., Roswandi N.L., Waqas M., Habib H., Hussain F., Khan S., Sohail M., Ramli N.A., Thu H.E., Hussain Z. (2018). Hyaluronic acid, a promising skin rejuvenating biomedicine: A review of recent updates and pre-clinical and clinical investigations on cosmetic and nutricosmetic effects. Int. J. Biol. Macromol..

[32] Janarthanan G., Shin H.S., Kim I.G., Ji P., Chung E.J., Lee C., Noh I. (2020). Self-crosslinking hyaluronic acid-carboxymethylcellulose hydrogel enhances multilayered 3D-printed construct shape integrity and mechanical stability for soft tissue engineering. Biofabrication.

[33] Li L., Wang N., Jin X., Deng R., Nie S., Sun L., Wu Q., Wei Y., Gong C. (2014). Biodegradable and injectable in situ cross-linking chitosan-hyaluronic acid based hydrogels for postoperative adhesion prevention. Biomaterials.

[34] Tokatlian T., Cam C., Segura T. (2015). Porous hyaluronic acid hydrogels for localized nonviral dna delivery in a diabetic wound healing model. Adv. Healthc. Mater..

[35] Tokatlian T., Cam C., Segura T. (2014). Non-viral DNA delivery from porous hyaluronic acid hydrogels in mice. Biomaterials.

[36] Laowpanitchakorn P., Zeng J., Piantino M., Uchida K., Katsuyama M., Matsusaki M. (2024). Biofabrication of engineered blood vessels for biomedical applications. Sci. Technol. Adv. Mater..

[37] Cui H., Zhu W., Nowicki M., Zhou X., Khademhosseini A., Zhang L.G. (2016). Hierarchical Fabrication of Engineered Vascularized Bone Biphasic Constructs via Dual 3D Bioprinting: Integrating Regional Bioactive Factors into Architectural Design. Adv. Healthc. Mater..

[38] Termeer C.C., Hennies J., Voith U., Ahrens T., Weiss J.M., Prehm P., Simon J.C. (2000). Oligosaccharides of hyaluronan are potent activators of dendritic cells. J. Immunol..

[39] Slevin M., Kumar S., Gaffney J. (2002). Angiogenic oligosaccharides of hyaluronan induce multiple signaling pathways affecting vascular endothelial cell mitogenic and wound healing responses. J. Biol. Chem..

[40] Zhao W., Li Y., Zhang X., Zhang R., Hu Y., Boyer C., Xu F.-J. (2020). Photo-responsive supramolecular hyaluronic acid hydrogels for accelerated wound healing. J. Control. Release.

[41] Xu Y., Han J., Chai Y., Yuan S., Lin H., Zhang X. (2018). Development of porous chitosan/tripolyphosphate scaffolds with tunable uncross-linking primary amine content for bone tissue engineering. Mater. Sci. Eng. C.

[42] Chen S., Cui S., Zhang H., Pei X., Hu J., Zhou Y., Liu Y. (2018). Cross-Linked Pectin Nanofibers with Enhanced Cell Adhesion. Biomacromolecules.

[43] Qu C., Bao Z., Zhang X., Wang Z., Ren J., Zhou Z., Tian M., Cheng X., Chen X., Feng C. (2019). A thermosensitive RGD-modified hydroxybutyl chitosan hydrogel as a 3D scaffold for BMSCs culture on keloid treatment. Int. J. Biol. Macromol..

[44] Park K.M., Joung Y.K., Park K.D., Lee S.Y., Lee M.C. (2008). RGD-Conjugated chitosan-pluronic hydrogels as a cell supported scaffold for articular cartilage regeneration. Macromol. Res..

[45] Kritchenkov A.S., Egorov A.R., Abramovich R.A., Kurliuk A.V., Shakola T.V., Kultyshkina E.K., Meza M.J.B., Pavlova A.V., Suchkova E.P., Thuy G.L.N. (2021). Water-soluble triazole chitin derivative and its based nanoparticles: Synthesis, characterization, catalytic and antibacterial properties. Carbohydr. Polym..

[46] Li X., Jin J., Xu W., Wang M., Liu L. (2022). Abortive ligation intermediate blocks seamless repair of double-stranded breaks. Int. J. Biol. Macromol..

[47] Arıcı Ş., Kamali A.R., Ege D. (2024). CMC/Gel/GO 3D-printed cardiac patches: GO and CMC improve flexibility and promote H9C2 cell proliferation, while EDC/NHS enhances stability. Biofabrication.

[48] Hua J., Li Z., Xia W., Yang N., Gong J., Zhang J., Qiao C. (2016). Preparation and properties of EDC/NHS mediated crosslinking poly (gamma-glutamic acid)/epsilon-polylysine hydrogels. Mater. Sci. Eng. C.

[49] Cui L., Li J., Guan S., Zhang K., Zhang K., Li J. (2022). Injectable multifunctional CMC/HA-DA hydrogel for repairing skin injury. Mater. Today Bio.

[50] Su C., Liu J., Yang Z., Jiang L., Liu X., Shao W. (2020). UV-mediated synthesis of carboxymethyl cellulose/poly-N-isopropylacrylamide composite hydrogels with triple stimuli-responsive swelling performances. Int. J. Biol. Macromol..

[51] Zhu H., Chen S., Duan H., He J., Luo Y. (2023). Removal of anionic and cationic dyes using porous chitosan/carboxymethyl cellulose-PEG hydrogels: Optimization, adsorption kinetics, isotherm and thermodynamics studies. Int. J. Biol. Macromol..

[52] Doane T.L., Chuang C.H., Hill R.J., Burda C. (2012). Nanoparticle ζ-potentials. Acc. Chem. Res..

[53] Paknia S., Izadi Z., Moosaipour M., Moradi S., Khalilzadeh B., Jaymand M., Samadian H. (2022). Fabrication and characterization of electroconductive/osteoconductive hydrogel nanocomposite based on poly(dopamine-co-aniline) containing calcium phosphate nanoparticles. J. Mol. Liq..

[54] Chen Y., Sun P. (2019). pH-Sensitive Polyampholyte Microgels of Poly(Acrylic Acid-*co*-Vinylamine) as Injectable Hydrogel for Controlled Drug Release. Polymers.

[55] Manamoongmongkol K., Sriprom P., Narkrugsa W., Phumjan L., Permana L., Kaewbutra S., Assawasaengrat P. (2024). Study on chemical structure stability and properties of chitosan-incorporated tamarind seed kernel xyloglucan hydrogels. Colloids Surf. A Physicochem. Eng. Asp..

[56] Neto C.D.T., Giacometti J.A., Job A.E., Ferreira F.C., Fonseca J.L.C., Pereira M.R. (2005). Thermal Analysis of Chitosan Based Networks. Carbohydr. Polym..

[57] Moradi L., Witek L., Nayak V.V., Pereira A.C., Kim E., Good J., Liu C.-J. (2023). Injectable hydrogel for sustained delivery of progranulin derivative Atsttrin in treating diabetic fracture healing. Biomaterials.

[58] Zhao Y., Liu X., Peng X., Zheng Y., Cheng Z., Sun S., Ding Q., Liu W., Ding C. (2022). A poloxamer/hyaluronic acid/chitosan-based thermosensitive hydrogel that releases dihydromyricetin to promote wound healing. Int. J. Biol. Macromol..

[59] Santhamoorthy M., Kim S.C. (2025). A Review of the Development of Biopolymer Hydrogel-Based Scaffold Materials for Drug Delivery and Tissue Engineering Applications. Gels.

[60] Kanikireddy V., Varaprasad K., Jayaramudu T., Karthikeyan C., Sadiku R. (2020). Carboxymethyl cellulose-based materials for infection control and wound healing: A review. Int. J. Biol. Macromol..

[61] Pessanha F.S., de Oliveira B.G.R.B., Oliveira B.C., Deutsch G., Teixeira F.L., Bokehi L.C., Calomino M.A., de Castilho S.R., Thiré R.M.d.S.M., Teixeira L.A. (2023). Effectiveness of Epidermal Growth Factor Loaded Carboxymethylcellulose (EGF-CMC) Hydrogel in Biofilm Formation in Wounds of Diabetic Patients: A Randomized Clinical Trial. Gels.

[62] Fan F., Saha S., Hanjaya-Putra D. (2021). Biomimetic Hydrogels to Promote Wound Healing. Front. Bioeng. Biotechnol..

[63] Martorana A., Lenzuni M., Contardi M., Palumbo F.S., Cataldo S., Pettignano A., Catania V., Schillaci D., Summa M., Athanassiou A. (2024). Schiff Base-Based Hydrogel Embedded with In Situ Generated Silver Nanoparticles Capped by a Hyaluronic Acid-Diethylenetriamine Derivative for Wound Healing Application. ACS Appl. Mater. Interfaces.

